# A novel cell-free intrathecal approach with PRP for the treatment of spinal cord multiple sclerosis in cats

**DOI:** 10.1186/s41232-022-00230-w

**Published:** 2022-10-14

**Authors:** Mariam F. Farid, Yara S. Abouelela, Noha A. E. Yasin, Mohamed R. Mousa, Marwa A. Ibrahim, Abdelbary Prince, Hamdy Rizk

**Affiliations:** 1 Anatomy and Embryology Department, Faculty of Veterinary Medicine, Cairo University, Giza, 12211 Egypt; 2 Cytology and Histology Department, Faculty of Veterinary Medicine, Cairo University, Giza, Egypt; 3 Pathology Department, Faculty of Veterinary Medicine, Cairo University, Giza, Egypt; 4 Department of Biochemistry and Molecular Biology, Faculty of Veterinary Medicine, Cairo University, Giza, Egypt; 5 Department of Biomedical Research, Armed Forces College of Medicine, Cairo, Egypt

**Keywords:** Multiple sclerosis, Platelet-rich plasma, BBB score, Demyelination, Ethidium bromide

## Abstract

**Background:**

Multiple sclerosis (MS) is a progressive autoimmune demyelinating disease of the central nervous system. To date, there is no effective therapy for it. Our study aimed to determine the potential role of platelet-rich plasma (PRP) in the treatment of MS in cats.

**Methods:**

The current study was conducted on 15 adult Persian cats that were divided into three groups: control negative, control positive (ethidium bromide (EB)-treated group), and PRP co-treated group (EB-treated group intrathecally injected with PRP on day 14 post-spinal cord injury). PRP was obtained by centrifuging blood on anticoagulant citrate dextrose and activating it with red and green laser diodes. The Basso–Beattie–Bresnahan (BBB) scores were used to assess the motor function recovery on days 1, 3, 7, 14, 20, and 28 following 14 days from EB injection. Moreover, magnetic resonance imaging (MRI) analysis, histopathological investigations, transmission electron microscopy (TEM) studies, and immunohistochemical analysis were conducted, and the gene expressions of nerve growth factors (NGFs), brain-derived neurotrophic factors (BDNF), and stromal cell-derived factors (SDF) were evaluated.

**Results:**

Our results indicated that PRP had a significant ameliorative effect on the motor function of the hindlimbs as early as day 20 and so on. MRI revealed that the size and intensity of the lesion were significantly reduced in the PRP co-treated group. The histopathological and TEM investigations demonstrated that the PRP co-treated group had a significant improvement in the structure and organization of the white matter, as well as a high remyelination capacity. Furthermore, a significant increase in myelin basic protein and Olig2 immunoreactivity as well as a reduction in Bax and glial fibrillar acidic protein immune markers was observed. NGFs were found to be upregulated by gene expression.

**Conclusion:**

As a result, we concluded that the intrathecal injection of PRP was an effective, safe, and promising method for the treatment of MS.

## Background

Multiple sclerosis (MS) is defined as a progressive, chronic, inflammatory demyelinating syndrome of the central nervous system (CNS) mediated by the immune system [1]. The immune system attacks the myelin sheath surrounding the axons. To date, the etiology of MS remains unidentified, thus resulting in various physical and mental disabilities [1–3]. The most important process in MS and other demyelinating diseases is CNS remyelination, which protects nerve fibers and restores functional recovery [4]. Previous studies have reported that spinal cord recovery after an injury is hampered by the formation of glial and myelin scars and inadequate trophic factors [5–7].

MS destroys oligodendrocytes, which are responsible for the production and maintenance of the myelin sheath, resulting in the thinning or complete loss of myelin [2], paralysis of the limbs, uncontrolled urination and defecation, muscle weakness, chronic pain, decreased tail movement, and visual, sensation, and sphincter problems [8].

Animal models have proven to be extremely useful in understanding the mechanism of the disease and the possible therapeutic interventions [9–11]. Cats, like other animals, suffer from a variety of neurological disorders, including the consequences of traumas, inflammations, congenital malformations, and infections. Also, paramyxovirus infection is considered an etiology for demyelination in cats [12]. Abdallah et al. [13] demonstrated that intraspinal injection of ethidium bromide (EB), as a gliotoxin, in the lateral columns of dogs resulted in progressive clinical disability, presence of sclerotic plaques by magnetic resonance imaging (MRI), severe histopathological lesions, axonal degeneration, and demyelination. Furthermore, [8] showed that EB significantly increased the immune expression of pro-apoptotic markers, Bax, and caspase 9, as well as glial fibrillary acidic protein (GFAP), and significantly decreased oligodendrocyte transcription factor (Olig2) and myelin basic protein (MBA).

Platelet-rich plasma (PRP) is a new generation of biological products that could be defined as a small proportion of plasma containing more platelets than the entire blood [14]. The platelet-rich portion contains over 20 different types of growth factors and cytokines. These factors, such as platelet growth factor, transforming growth factor, insulin-like growth factor, endothelial growth factor, coagulation factor, granulocyte-macrophage colony-stimulating factor, hepatocyte growth factor, and epithelial growth factor that supports wound healing, modify the extracellular matrix and promote cell proliferation and angiogenesis [15–18]. In regenerative medicine, PRP has remarkable healing abilities for muscle disorders [19], bone diseases and wounds [20], osteochondral defect [21], tendon disorders [22, 23], osteoarthritis [24–26], brain injuries [27], spinal cord injury [28], and burns [29].

Despite its wide range of applications, little attention has been paid to PRP’s potential therapeutic effect in MS models. Thus, this study aimed to assess the effectiveness of PRP in the treatment of MS by evaluating the improvement in the cases via anatomical dissection, MRI, Basso–Beattie–Bresnahan (BBB) score, histopathology, transmission electron microscopy (TEM), immunohistochemistry, and gene expression.

## Materials and methods

### Chemicals and reagents

EB was purchased from Suvchem Laboratory Chemicals. Xylazine was obtained from Xyla-Ject® 2% ADWIA Co., A.R.E. and ketamine 5% from Ketamar® 5% Sol. Amoun Co. A.R.E. Sodium thiopental was purchased from Thiopental® EPICO, A.R.E., Phosphate buffer saline® Sigma, Aldrich.

### Animals

All animals were treated and used by following ethical approval from the Veterinary Medicine Cairo University Institutional Animal Care and Use Committee (Vet-CU-IACUC) with approval number Vet Cu12/10/2021/393. A total of 15 male adult Persian cats (2–3 years) apparently healthy were collected from pet shops and shelters around Giza and housed at the Faculty of Veterinary Medicine. The cats were fed a cat-specific diet and water ad libitum with a 12/12 light–dark cycle. They were subjected to spinal cord injury after 1 week of acclimatization and continuous observation for any nervous manifestations.

### Experimental design

**Fig. 1:**
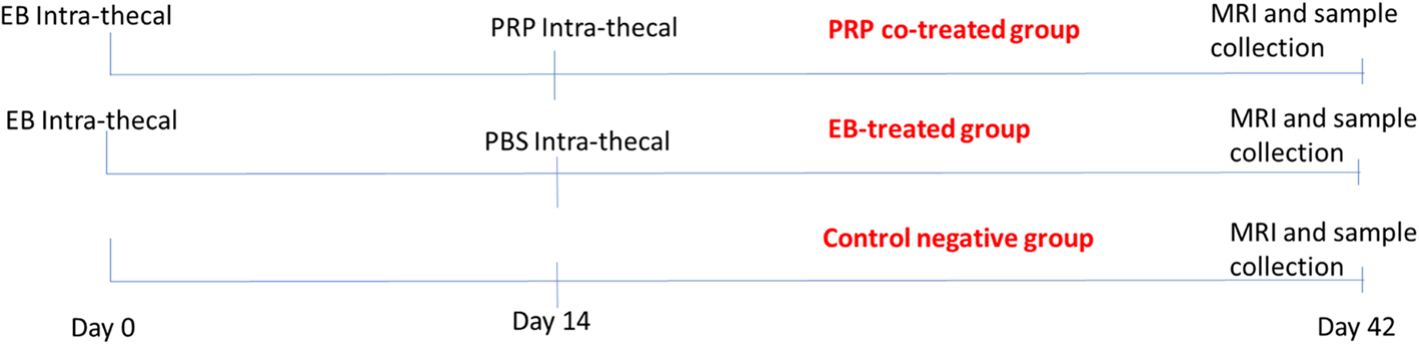
Diagram showing the timeline of the treatment procedure

The study compared three groups of thoraco-lumbar MS (*n* = 15). Group I (control negative) (*n* = 5) received no injury induction or treatment. Spinal cord injury was induced using EB in group II (control positive or EB-treated group) (*n* = 5); the animals in this group received a single injection of 1-mL phosphate-buffered saline (PBS) intrathecally into the foramen magnum. In group III (PRP co-treated group) (*n* = 5), spinal cord injury was induced, and the animals in this group received 1-mL laser-activated PRP intrathecally into the foramen magnum (Fig. 1).

#### Spinal cord demyelination

The spinal cord of the cats in groups II and III were demyelinated using EB [30–32]. The cats were anesthetized with xylazine (IM, 1 mg/kg) and ketamine 5% (IV, 10 mg/kg) and then placed on sternal recumbency; from T12 to L2, a dorsal midline incision was made. After dissecting the subcutaneous fascia and lumbosacral fascia, a supraspinous ligament incision was made along the spinous process and midline. Subsequently, the multifidus lumborum was dissected bluntly to reach the dorsal lamina of L1. Using a dental drill of round bur 2, bilateral holes were created and 6 μL from EB 0.1% W/V solution in PBS was injected into each hole [33]. After that, the dorsal musculature and skin were sutured. Locomotor functions were monitored and documented using the BBB locomotion scale [34].

#### Preparation and activation of PRP

Under the effect of tranquilizer (xylazine 1 mg/kg, I/M), blood was collected from the jugular vein on anticoagulant citrate dextrose solution (1:9), manually prepared according to the method described by [35]. Then, the blood was centrifuged at 3000 rpm for 3 min, and the supernatant that consisted of plasma and buffy coat was collected and transferred to a new tube for further centrifugation at 4000 rpm for 15 min. After the second centrifugation, the upper third of the supernatant was formed by platelet-poor plasma, and the lower third was formed by PRP [7, 28]. The PRP was then collected in a tube containing 1-mL sterile PBS and activated by two laser diodes; one in the red and one in the green (635 and 516 nm, respectively) were applied at a distance of 10 cm and a power density of 80 mW/cm^2^ [36, 37].

#### Treatment with PRP

In group II, 1 mL of PBS was injected intrathecally into the foramen magnum on day 14 after demyelination. In group III, 1 mL of activated PRP in PBS was injected intrathecally into the foramen magnum on day 14 post-induction of spinal cord injury. To prevent fluid leakage, the needle was maintained for a few seconds (Fig. 1).

### Behavioral analysis

The locomotor function was assessed using the 21-point BBB locomotion scale at 1, 3, 7, and 14 days after demyelination induction as well as 1, 3, 10, 14, 20, and 28 days after treatment.

### Magnetic resonance imaging (MRI)

Under general anesthesia, MRI was performed 28 days after receiving PRP or PBS treatment using ECHELON Smart (Hitachi 1.5T Supercon MRI, Japan). The imaging protocol was performed on T11 to L3. The procedure included transverse T2-weighted (TR/TE 3290/99 ms) and T1-weighted (TR/TE 651/12 ms) and sagittal STIR (TR/TE/TI 3310/61/140 ms) sequences, as well as sagittal and dorsal T2-weighted (TR/TE 2880/111 ms) and T1-weighted (TR/TE 623/1 ms).

Subsequently, all animals were anesthetized with xylazine (1 mg/kg, IM) and ketamine (5% IV 10 mg/kg); then, they were euthanized by sodium thiopental injection at a lethal dose of 67 mg/kg, IV [38].

### Gross morphology of the spinal cord

A dorsal midline incision was made at the T11–L3 level; then, the fascia and back muscles were dissected until the spinous processes and vertebral bodies were reached. The spinous process was carefully cut using a bone cutter; then, the spinal cord was extracted from the vertebrae for morphological assessment.

### Histopathology

#### Light microscopy

All groups had their spinal cord tissue specimens collected and fixed in 10% neutral buffered formalin. Tissue specimens were routinely processed in alcohols and xylenes, embedded in melted paraffin wax, and cut into 5-μm sections. For light microscopy, hematoxylin and eosin staining protocols were adopted [39]. An Olympus BX43 microscope (Olympus, Tokyo, Japan) was used to examine the stained slides and an Olympus Dp27 digital camera (Olympus, Tokyo, Japan) to capture the images.

#### Transmission electron microscopy (TEM)

Small specimens of the spinal cord, approximately 1 mm, obtained from all groups were fixed in 3% glutaraldehyde in 0.1 M phosphate buffer for a few hours and then post-fixed in 1% osmium tetroxide for 1 h. Subsequently, the 1-μm-thick semi-thin sections were stained with toluidine blue. The selected areas were cut into ultrathin sections of approximately 50 nm and then stained with uranyl acetate and lead citrate. Finally, the sections were examined via TEM (TEM-109, SEO Company) in the Electron Microscopy Unit, Faculty of Agriculture, Cairo University, Egypt.

#### Immunohistochemistry

Paraffin-embedded blocks were used to obtain several tissue sections that were fixed on adhesive slides for immune staining. Endogenous peroxidase and protein blocking steps were performed after rehydration using hydrogen peroxide and bovine serum albumin (BSA). The tissue slides were washed with Tris buffer several times, followed by the application of primary antibodies (anti-MBP sc-271524, anti-GFAP sc-33673, anti-Olig2 sc-293163, and anti-Bax sc-7480, Santa Cruz Biotechnology, Inc., Heidelberg, Germany) at a dilution of 1:150 for 12 h at 4 °C. After that, washing was done for 2 h at room temperature with a secondary horseradish peroxidase (HRP)-labeled antibody (goat anti-mouse HRP-labeled secondary antibody, Abcam, UK) at a dilution of 1:1000. The color was developed using the DAB Substrate Kit. Negative control slides were obtained by skipping the primary antibody step. Positive expression was quantified as area % using cellSens dimensions (Olympus Software).

### Gene expression

Total RNA was isolated by using the easy-spin Total RNA Extraction Kit (iNtRON Biotechnology DR, Cat. No.17221) according to the manufacturer’s protocols [40]. The RNA quality and quantity were assessed using a NanoDrop ND-1000 spectrophotometer (NanoDrop Technologies), and the total RNA of each sample was about 8 μg/sample. The cDNA was generated using M-MuLV Reverse Transcriptase (NEB#M0253) according to the provided protocol. Real-time reverse transcription-polymerase chain reaction (RT-PCR) was used to analyze the expression of target genes, and the mRNA levels were detected using the HERA^PLUS^ SYBR Green qPCR Kit (#: WF10308002). The primer sets are presented in Table 1. The cycle conditions were as follows: 95 °C for 2 min and 40 cycles of 95 °C for 10 s and 60 °C for 30 s. Each RT-PCR was conducted in triplicate. Glyceraldehyde 3-phosphate dehydrogenase was used as an internal control [41]. The data obtained from the qRT-PCR were analyzed using CT, ΔCT, ΔΔCT, and 2^−ΔΔCT^ [42].

**Table 1:** Primers sequences used for qRT-PCR

Gene symbol	Primer sequence
BDNF	Forward primer: 5′-CGGTCACCGTCCTTGAAAA-3′ Reverse primer: 5′-GGATTGCACTTGGTCTCGTAGAA-3′
GAPDH	Forward primer: 5′-TGGAAAGCCCATCACCATCT3-′ Reverse primer: 5′-CAACATACTCAGCACCAG CATCA-3′
SDF1	Forward primer: 5′–ACAGATGTCCTTGCCGATTC–3 Reverse primer: 5′-CCACTTCAATTTCGGGTCAA–3
NGF	Forward primer: GCAGGGCAGACCCGCAACAT Reverse primer: GCACCACCCGCCTCCAAGTC

### Statistical analysis

Three replicates per group was used, and the data from complete random samples were subjected to two-way analysis of variance (ANOVA) for gait score analysis. When *P* ≤ 0.05, Fisher’s exact test was used to compare the treatments. Letters a–i were used on the columns to express the significant difference between the groups and days of the experiment. Pearson’s correlation coefficient was executed using the OriginPro statistical software package version 2016. The data were analyzed using GraphPad Prism version 8.4.3 (686) in one-way ANOVA and *P* < 0.05 in gene expression analysis and immunohistochemistry [40].

## Results

### Gross morphology of the spinal cord

**Fig. 2:**
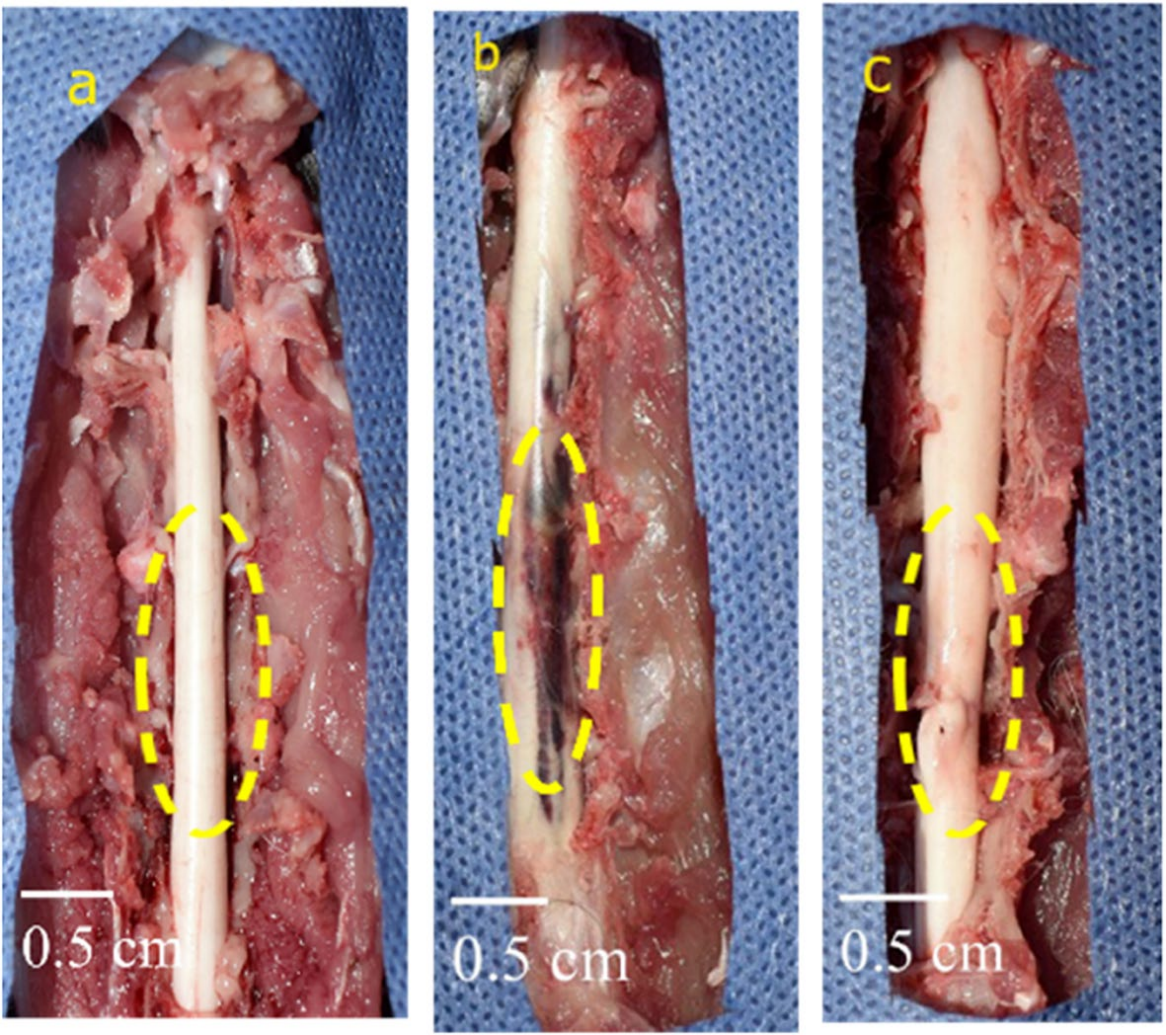
The gross morphology of the spinal cord at the end of the experiment at day 42. **A** Control negative group. **B** EB-treated group. **C** PRP co-treated group

The spinal cord length from T11 to L3 ranged from 6 to 6.5 cm. In the control negative group, the spinal cord appeared as a whitish long cylindrical tube surrounded by the dura matter with no abnormalities. On the other hand, in the EB-treated group, the cord was characterized by the presence of a distinct reddish-brown hemorrhagic sizable area at the T12–L1 level within the spinal cord structure (intramedullary). The spinal cord appeared as a whitish tube with a small focal brown lesion in the PRP co-treated group (Fig. 2).

### Gait score analysis

All cats in the three groups had a BBB score of 21 before the injury induction, and the cats were monitored at 1, 3, 7, and 14 days after the demyelination induction; their gait score was recorded using the BBB gait score analysis for the hindlimb movement.

Eight cats from groups II and III demonstrated a considerable loss of hindlimb motor function, and the BBB score decreased to 0 or 1 and gradually improved to 3 by day 14. Although only two cats from these groups showed improper walking steps, incoordination between the forelimbs and hindlimbs, and ataxia only, therefore, they were excluded from the study.

**Fig. 3:**
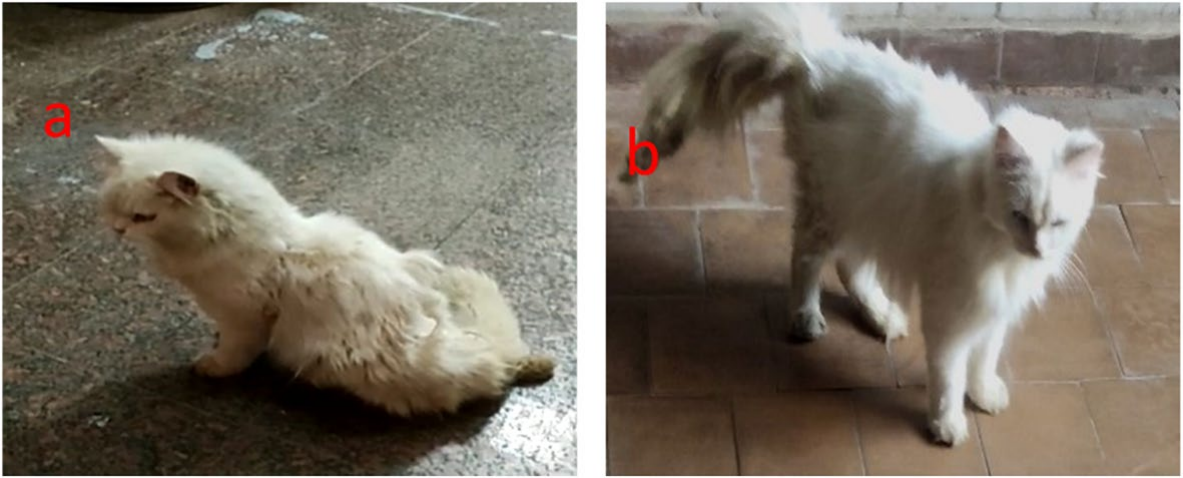
The effect of treatment on motor function in PRP co-treated group. **A** After induction of injury. **B** After receiving PRP as treatment

**Fig. 4:**
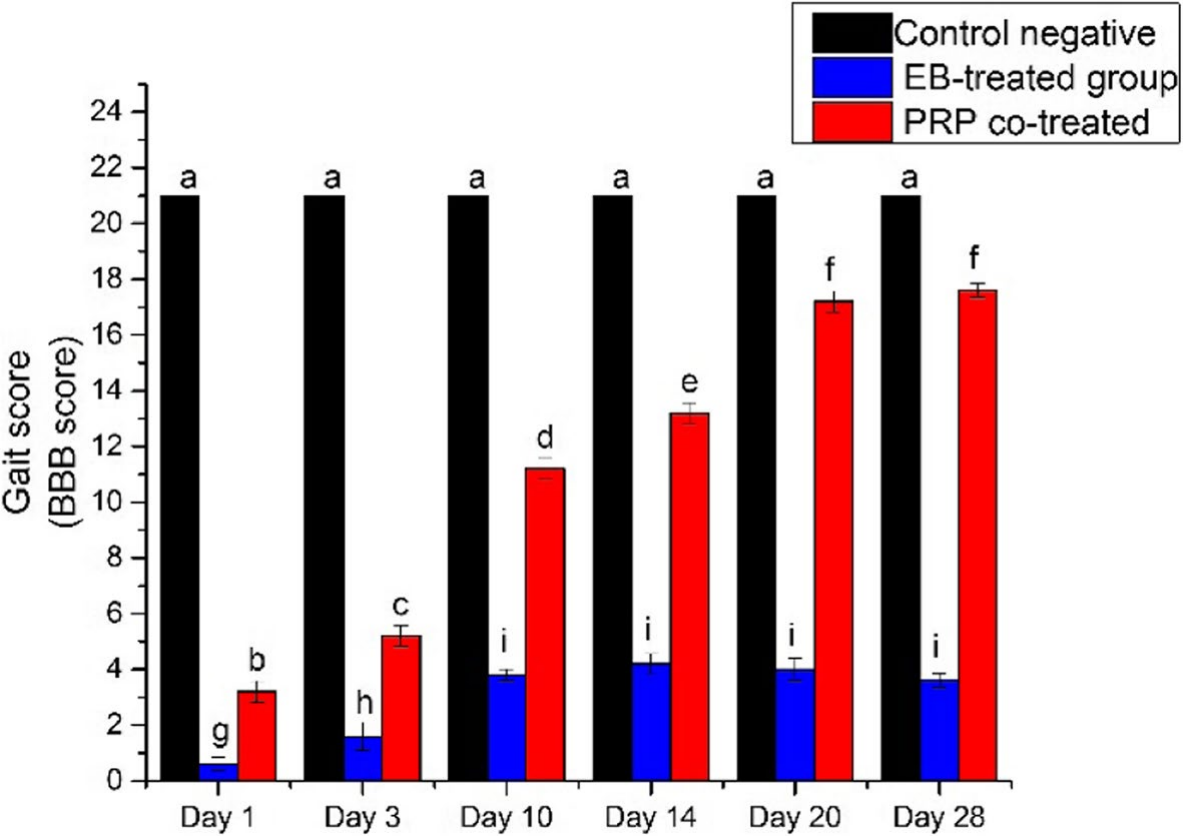
Gait score (BBB score) during the study. The gait score of the cats of the PRP co-treated group was significantly increased over time (red column) except between days 20 and 28 where *P* ≤ 0.05. The gait score of the control negative group (black column) showed no significant differences all over the experiment, and the EB-treated group (blue column) showed significant differences between days 1 and 3 but no significant difference after. The columns of the same letter have no significance difference in between (for the interpretation of the references to color in this figure legend, the reader is referred to the web version of this article)

A significant difference in the gait BBB score was observed between the PRP co-treated group and other groups. Except between days 20 and 28, when the animals reached a BBB score of 18, the PRP co-treated group exhibited a significant increase in BBB score with time except between days 20 and 28 where animals reached 18 points on BBB score (Fig. 3). In the control group, the BBB score did not significantly differ between the days of the experiment. The EB-treated group showed a significant difference on days 1, 3, and 10, but none after that, with the maximum score being 5 (Fig. 4).

### MRI

**Fig. 5:**
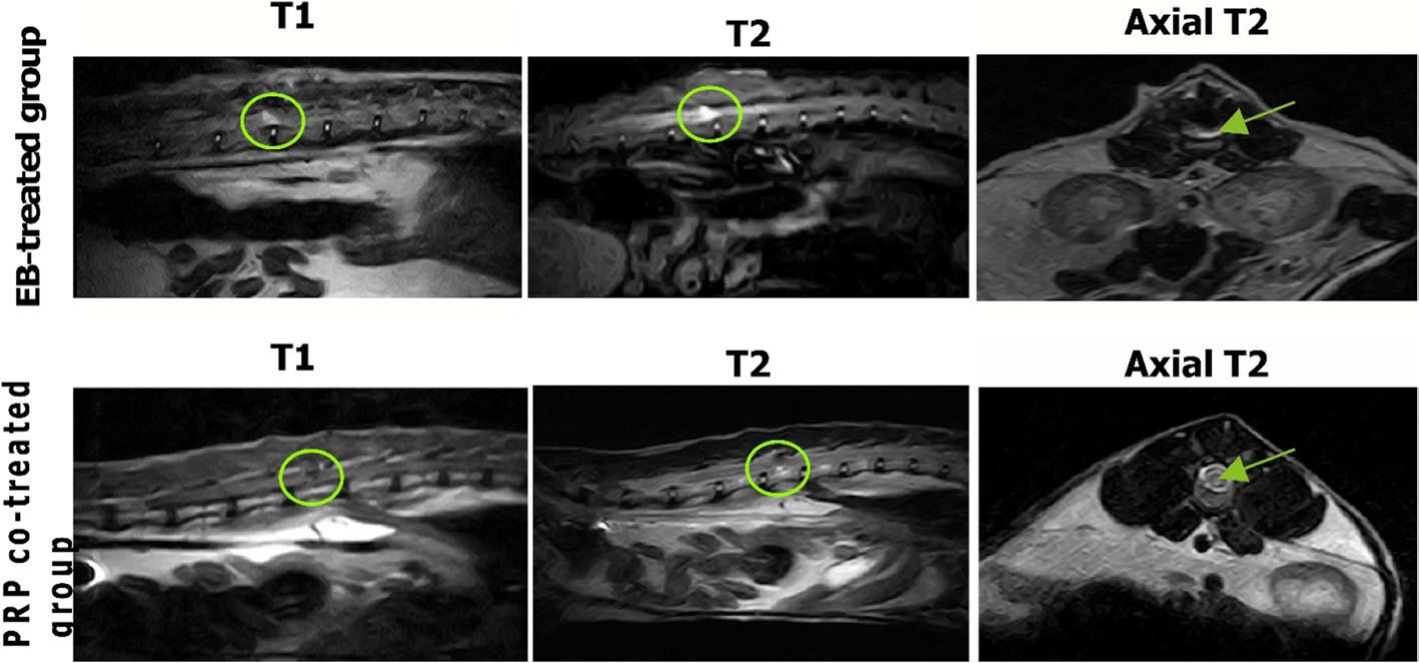
MRI analysis of the spinal cord. The EB-treated group is characterized by a large hypointense lesion (circle) on sagittal T1 scan and a diffuse hyperintense lesion on sagittal (circle) and axial (arrow) T2 scans while the PRP co-treated group has a small faint hypointense lesion on sagittal T1 scan and a faint hyperintense lesion on sagittal and axial T2 scans showing decreased intensity and extent of the lesion (circle and arrow)

To determine the extent and location of the injury, MRI analysis was conducted. At the T13–L1 level, the cats in the EB-treated group had a diffuse hyperintense lesion on the sagittal and axial T2-weighted images and hypointense lesion on the sagittal T1-weighted ones, indicating the presence of sclerotic plaque, while the PRP co-treated group showed smaller faint lesions on the T2, T1 sagittal, and axial-weighted images. When compared with the EB-treated group, the extent of the injury in the PRP co-treated group was significantly reduced (Fig. 5).

### Histopathology

#### Light microscopy

**Fig. 6:**
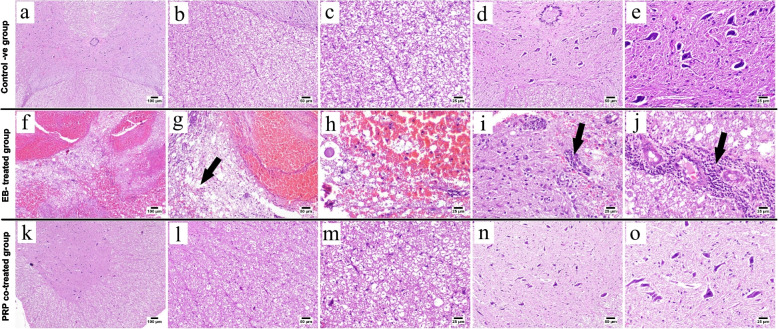
Photomicrograph of H&E-stained spinal cord sections. **a**–**e** Control negative group showing normal histology of white matter (**b**, **c**) and gray matter (**d**, **e**). **f**–**j** EB-treated group showing **f** diffuse hemorrhage in the spinal cord; **g** hemorrhage with marked demyelination (arrow); **h** higher magnification, hemorrhage with vacuolated areas of demyelination; **i** gliosis (arrow); **j** marked perivascular lymphocytic infiltration. **k**–**o** PRP co-treated group showing **k** apparently normal spinal cord, **l**, **m** apparently normal white matter, and **n**, **o** apparently normal gray matter

Microscopic examination of the spinal cord obtained from the control negative group (Fig. 6a–e) revealed a normal structure of the spinal cord; both the white and gray matters were histologically normal. The spinal cord obtained from the EB-treated group (Fig. 6f–j) showed a wide spectrum of histopathological alterations, including extensive diffuse hemorrhage occupying both the white and gray matters. Wide vacuolated areas were scattered within the white matter, indicating nerve fiber demyelination. There was also evidence of axon degeneration. Gliosis, chromatolysis, and neuronal degeneration were observed in the gray matter. Perivascular lymphocytic infiltrations were also frequently seen. Conversely, the PRP co-treated group (Fig. 6k–o) showed minimal changes such as mild demyelination in the white matter of the spinal cord, whereas the majority of the examined sections were normal.

#### TEM

**Fig. 7:**
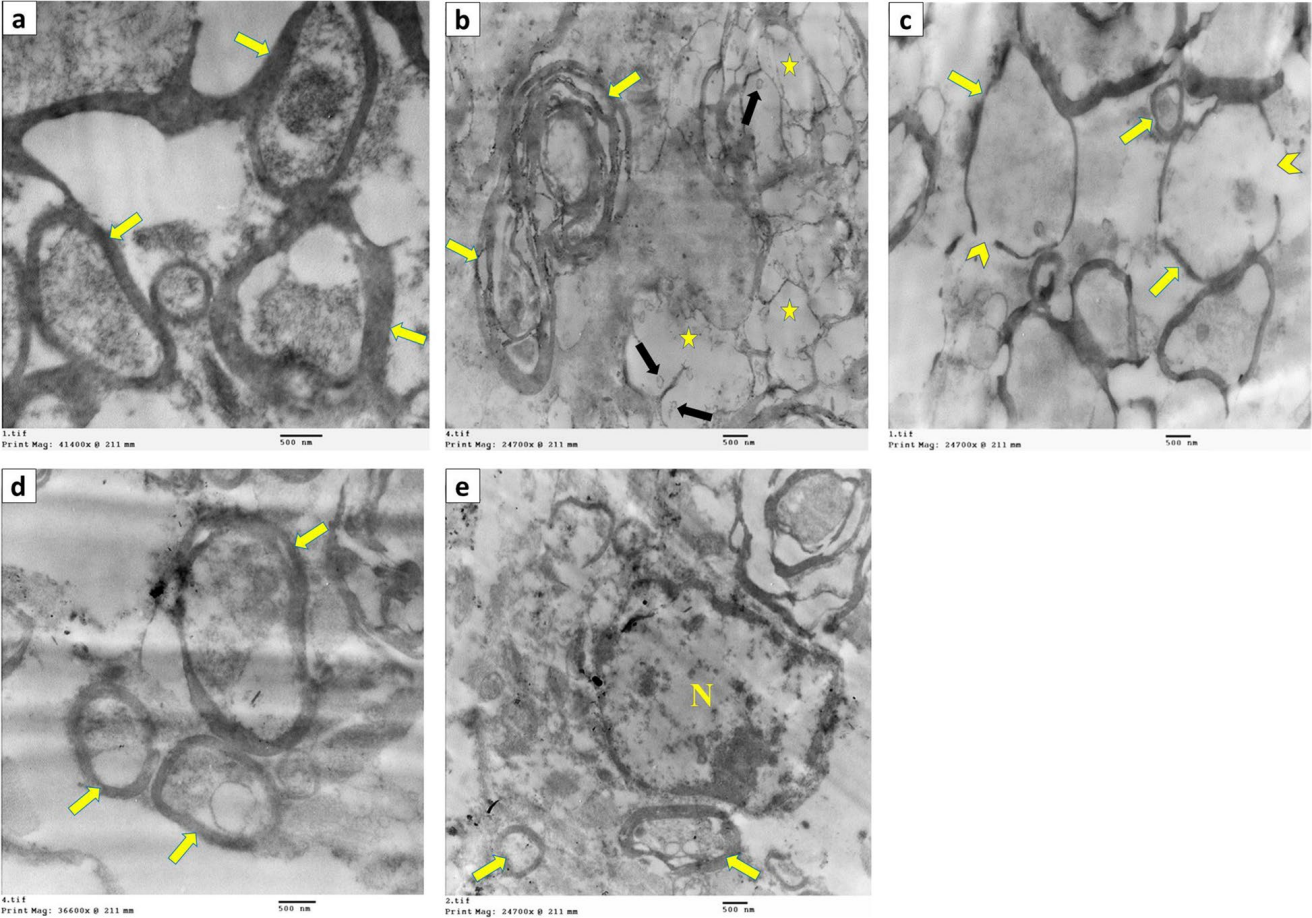
Electron micrograph of the spinal cord sections demonstrating **a** control group with intact dense compact myelin sheath (arrow) (× 20,000). **b**, **c** EB-treated group **b** exhibiting areas of demyelination and axonal swelling (star), degenerated mitochondria with complete loss of cristae (black arrow). Moreover, the splitting of myelin lamellae giving an onion-like appearance with shrunken atrophied axons is obvious (yellow arrow) (× 10,000). **c** Some nerve fibers reveal thin (arrow) interrupted (chevron) myelin sheaths (× 10,000). **d**, **e** PRP co-treated group **d** displaying reconstructed structure with regular more compact and thicker myelin sheaths (arrow) (× 15,000) and **e** Showing oligodendrocyte with euchromatic nucleus (N) and high capacity of remyelination (arrow) (× 10,000)

Ultrastructural examination of the white matter of the spinal cord from the control group demonstrated nerve fibers with regular intact, dense, compact myelin sheaths (Fig. 7a). In contrast, the EB-treated group showed areas of demyelination, axonal swelling, and degeneration, as well as degenerated mitochondria with complete loss of cristae. Splitting of the myelin lamellae with shrunken atrophied axons was also found, showing an onion-like structure (Fig. 7b). Thin interrupted myelin sheaths were detected in other nerve fibers (Fig. 7c). The PRP co-treated group, on the other hand, had a reconstructed structure with regular, compact, and thicker myelin sheaths (Fig. 7d). Furthermore, oligodendrocytes with euchromatic nuclei and a high remyelination capacity were observed (Fig. 7e).

#### Immunohistochemistry

##### Olig2 expression

**Fig. 8:**
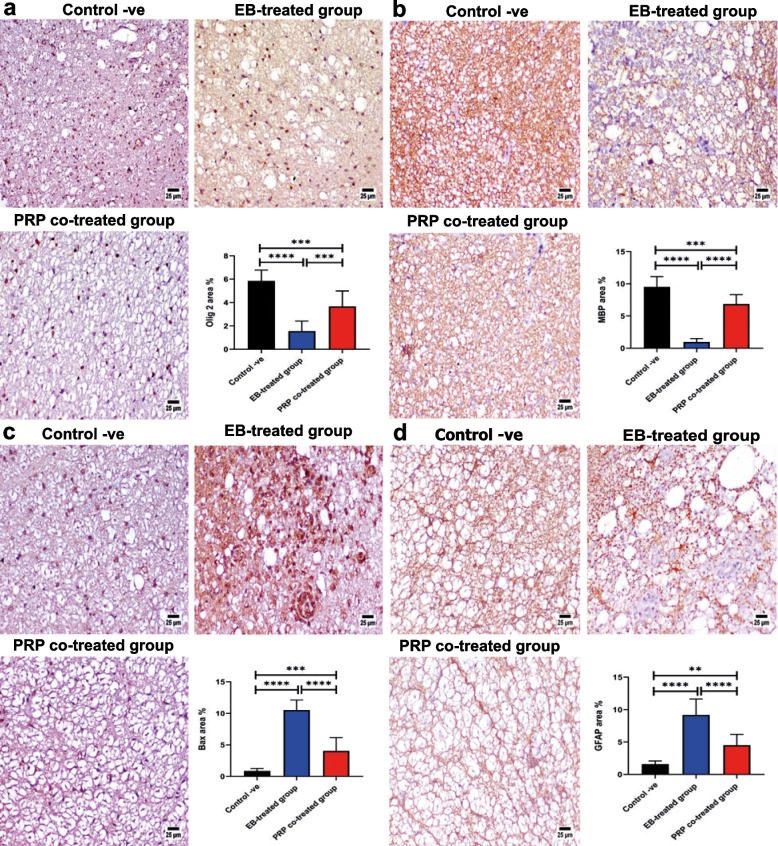
Photomicrographs of the spinal cord. **a** Olig2 expression; the EB-treated group reveals reduced immune expression with noticeable restored value in the PRP co-treated group. **b** MBP expression; the EB-treated group shows reduced expression with significant restored value in the PRP co-treated group. Charts showing the quantification of positive staining as area percent. Data were presented as means ± SE. A significant difference was considered at *P* < 0.05. **c** Bax expression exhibiting increased positive staining in the EB-treated group with a marked reduction in the PRP co-treated group. **d** GFAP expression showing increased positive immunostaining in the EB-treated group with a marked reduction in the PRP co-treated group. Charts showing the quantification of positive staining as area percent. Data were presented as means ± SE. A significant difference was considered at *P* < 0.05

A significantly higher Olig2 value was detected in the PRP co-treated group when compared with the EB-treated group (Fig. 8a).

##### MBP expression

The control negative group had a significantly higher value of MBP-positive expression (Fig. 8b), followed by the PRP co-treated group. The EB-treated group, on the other hand, showed a significant decrease in the MBP expression.

##### Bax expression

The positive expression of Bax was significantly increased in the EB-treated group when compared with the other experimental groups. In comparison with the EB-treated group, the PRP co-treated group showed a significant reduction in Bax-positive staining (Fig. 8c).

##### GFAP expression

Positive immune staining with GFAP (Fig. 8d) was significantly increased in the EB-treated group (control positive group) when compared with the other experimental groups. In comparison with the EB-treated group, there was a significant reduction in the GFAP expression in the PRP co-treated group.

### Gene expression analysis

**Fig. 9:**
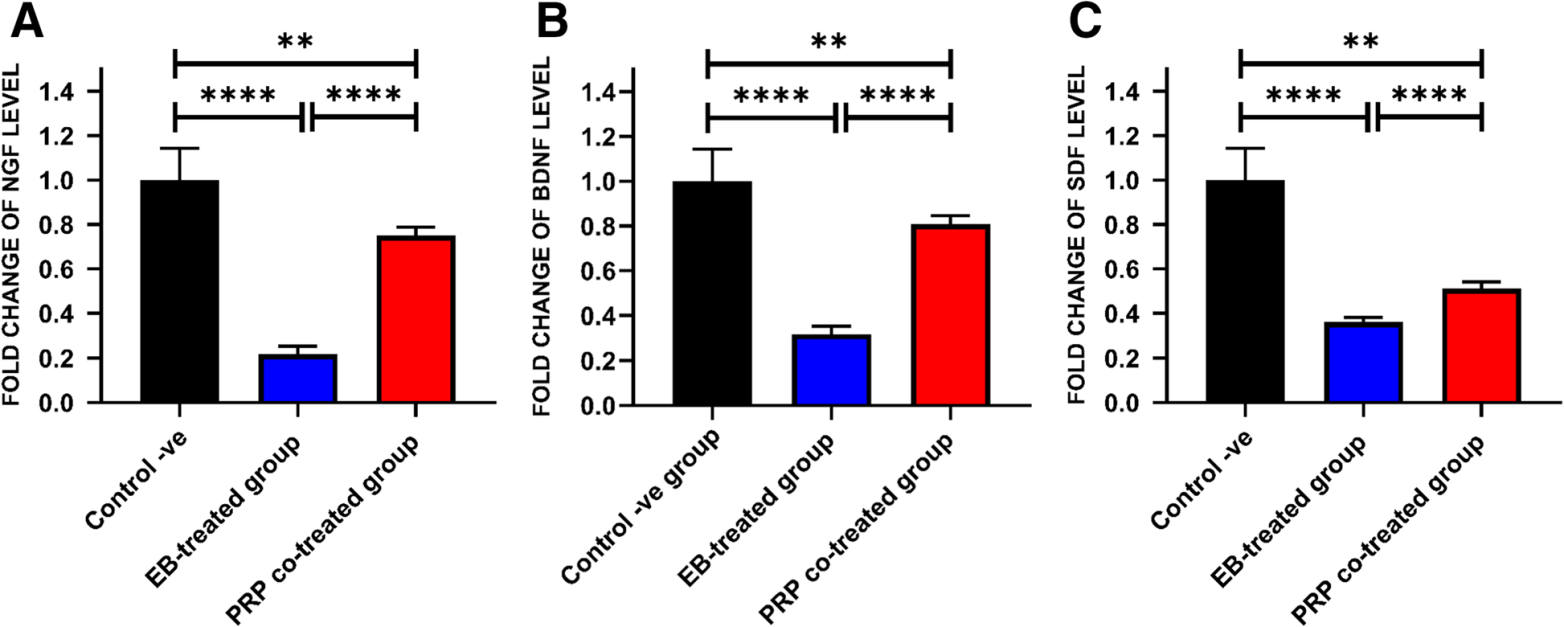
The relative expression levels of the target genes. **A** NGF transcript. **B** BDNF transcript. **C** SDF transcript. Data were presented as means ± SE. A significant difference was considered at *P* < 0.05

The expression levels of the neurotrophic factors, such as NGF and BDNF, the regulator of the remyelination process, neuronal regeneration, and neurotransmission, were higher in the control negative group than in the other groups. The expression levels of NGF and BDNF mRNAs were significantly higher (*P* < 0.05) in the PRP co-treated group than in the EB-treated group, indicating healthier tissue and higher regeneration activity. The transcription of chemokine SDF significantly increased (*P* < 0.05) in the PRP co-treated group compared with the EB-treated group, indicating neuronal regeneration in the area of demyelination (Fig. 9).

## Discussion

In this study, we investigated the effect of PRP on the SCI model in cats (MS). PRP improved different parameters, such as functional recovery, structural architecture, neuronal regeneration, remyelination, apoptosis regression, and gene expression. Some of the results were reported in different models of spinal cord injuries [7, 28]. On the other hand, PRP did not regenerate the sciatic nerve after injury according to [43]. Morishita et al. [44] found out that PRP did not affect knee arthroplasty in another study. The present work focused on the role of PRP on MS in the spinal cord, which had not been investigated previously, except for [45] in the treatment of encephalomyelitis MS in mice.

Demyelination caused by an intraspinal injection of gliotoxins, such as EB, which was used in our study, has a transient effect on the spinal cord resembling MS attacks with better treatment follow-up [46]. EB has been analyzed in animals such as rats, mice, cats, and dogs [13, 30, 31, 33, 47, 48] to gain insight into the causes of remyelination failure and to obtain a fuller knowledge of the axonal conduction disorders in MS. Our finding indicated that the EB resulted in hindlimb locomotion impairment. On the other hand, [49] demonstrated that no behavioral deficits were obtained using the BBB score in rats after injection with EB.

Our work shows that the intrathecal approach for the spinal cord is effective. Because MS is a multifocal disease, the treatment can be delivered to different sites in the spinal cord; this finding agrees with that of [50]. However, the lesional approach described by [51, 52] in the treatment of SCI may miss spots of MS.

Our clinical analysis of gait based on the BBB score was evident as early as the 10th day after treatment, with improvement in gait and proprioception reflexes that continued over time, and by the 28th day after treatment, the animals were able to walk normally with minimal locomotion impairment. Other studies have found that PRP therapy improves locomotor impairment in animals after spinal cord injury [7, 28].

The post-mortem findings in the current study indicated the presence of a large intramedullary hemorrhagic area in the EB-treated group. In the same area appeared small focal brown spot in the PRP co-treated group [50]; on the other hand, it is stated that there was no difference in the spinal cord morphologies between the different groups.

In our study, the existence of sclerotic plaque was revealed by MRI in the EB-treated group, which showed a diffuse hyperintense lesion on the sagittal and axial T2-weighted images and hypointense lesions on the sagittal T1-weighted ones. However, the T1-, T2-, and axial T2-weighted images in the PRP co-treated group revealed smaller and fainter lesions in the spinal cord, which is consistent with the findings of [8] in dogs with MS and of [53] in dogs with chronic spinal cord injury.

Our histopathological investigations revealed that the white matter of the EB-treated group showed extensive demyelination, severe axonal degeneration and vacuolation, and neuronal loss accompanied by an increasing number of astrocytes; these findings are consistent with those of previous studies [8, 13, 54–56]. Electron microscopy confirmed these findings, revealing the areas of demyelination, axonal swelling, and degeneration; degenerated mitochondria with complete loss of cristae; myelin splitting with shrunken atrophied axons; and thinning of the interrupted myelin sheath. These findings are consistent with those of [8, 13]. Mitochondrial disruption plays a critical role in neuronal dysfunction [57] and may lead to further axonal degeneration [58]. Axonal demyelination and interrupted myelin sheath interfere with nerve impulse transduction [50, 58], resulting in muscular weakness and impaired locomotor activities, as demonstrated by the current study. Demyelination induced by EB was confirmed by a significant decrease in the Olig2 and MBP immunoreactivities. The PRP co-treated group, on the other hand, showed minimal demyelination with a high remyelination capacity. This result was confirmed by significantly higher expressions of Olig2 and MBP, which are highly correlated with oligodendrocyte formation and remyelination, in the PRP co-treated group as compared with the EB-treated group. These results are consistent with those previously reported by [8, 45, 59] in different CNS demyelination models. In the same line, [7] demonstrated that receiving PRP 24 h after spinal cord injury can improve axonal regeneration and, as a result, cause functional motor recovery in rat models. MBP is responsible for maintaining the attachment of a multilayered compact myelin’s cytosolic surfaces [60, 61] demonstrating that the overexpression of Olig2 accelerates the generation of differentiated oligodendrocytes, resulting in neural tissue repair and precocious CNS myelination. Our results expand on the role of growth factors present in PRP in the treatment of neurodegenerative diseases.

In the present study, the immunohistochemistry analysis of the examined sections revealed significantly higher expressions of GFAP in the EB-treated group, which indicated increased gliosis and reactivity of astrocytes against MS, supporting the idea of using GFAP as a biomarker for MS reported by [62–64]. In the same context, the expression of GFAP was markedly reduced in the PRP co-treated group, as previously mentioned by [8, 45].

The protective role of the PRP growth factors on oligodendrocytes and neurons against Bax in neurological disorders was discussed by [45–66]. Our study recorded a significantly higher expression of Bax in the EB-treated group than in the control negative group, which is in line with the finding of [8] in MS [67]. Treatment with PRP, on the other hand, significantly reduced the high level of Bax expression in the EB-treated group. Salarinia et al. [68] observed that PRP can significantly suppress the upregulation of caspase 3, an apoptotic gene, and non-significantly retrieve the Bax and Bcl-2 gene expressions in the SCI rat model.

BDNF, a member of the neurotrophin gene family, contains NGF and neurotrophins 3 and 4 (NT3 and NT4) [69]. It plays a significant role in both healthy and diseased brains’ neuronal and oligodendroglial growth and survival. BDNF is a neurotrophic protein that is widely expressed in the CNS and has neuroprotective properties, such as neuronal survival, differentiation, axonal development, and myelination by activating its tyrosine receptor kinase (Trk) [70]. BDNF overexpression lowers axonal damage and alleviates symptoms [71]. BDNF is produced in the early stages of demyelination from the CNS-mediated axonal protective effects. According to these findings, mature BDNF had a neuroprotective impact during the progression of MS [72]. In the damaged CNS, BDNF affects the inflammatory homeostasis [73]. Immune cells express BDNF in actively demyelinating MS lesions but not in lesions where the myelin breakdown is not occurring [70]. PRP treatment promotes axonal remyelination due to BDNF overexpression [74]. Increases in neurotrophic factors, such as NGF and brain-derived neurotrophic factor, likely mediated these effects [75].

Stromal cell-derived factor-1 (SDF-1) is a pleiotropic chemokine that stimulates adaptive immune responses and angiogenesis in the bone marrow by attracting endothelial progenitor cells [76]. After an injury, a critical role seems to be played by BDNF, NGF, and SDF. Instead of having a neurotrophic effect as under normal conditions, the three mediators may induce hyperexcitability of injured neurons [77]. The upregulation of the three studied genes in this work might indicate the positive role played by the PRP in the regeneration of Ms.

## Conclusion

Our results indicated that a single injection of laser-activated PRP can significantly improve locomotion and sensory activities without impairing the CNS, enhance axonal regeneration, promote remyelination, inhibit apoptosis, improve angiogenesis and immune response, and alleviate the histopathological changes induced by EB. We concluded that PRP may have neuroprotective and neurotrophic effects in cats, resulting in a successful therapeutic effect in the treatment of neurodegenerative diseases and MS.

## Data Availability

All data collected or analyzed during this study are included in this published paper.

## Abbreviations

MS: Multiple sclerosis
PRP: Platelet-rich plasma
SDF: Stromal cell-derived factor
Oligo 2: Oligodendrocyte transcription factor
BBB: Basso–Beattie–Bresnahan
NGF: Nerve growth factor
MBA: Myelin basic protein
GFAP: Glial fibrillary acidic protein
BSA: Bovine serum albumin
HRP: Horseradish peroxidase
DAB: Diaminobenzidine
BDNF: Brain-derived neurotrophic factor
